# Structural model of FeoB, the iron transporter from *Pseudomonas aeruginosa*, predicts a cysteine lined, GTP-gated pore

**DOI:** 10.1042/BSR20160046

**Published:** 2016-04-27

**Authors:** Saeed Seyedmohammad, Natalia Alveal Fuentealba, Robert A.J. Marriott, Tom A. Goetze, J. Michael Edwardson, Nelson P. Barrera, Henrietta Venter

**Affiliations:** * Department of Pharmacology, University of Cambridge, Tennis Court Road, Cambridge CB2 1PD, U.K.; † Department of Physiology, Faculty of Biological Sciences, Pontificia Universidad Católica de Chile, Santiago, Chile; ‡ School of Pharmacy and Medical Sciences, Sansom Institute for Health Research, University of South Australia, GPO Box 2471, Adelaide 5001, Australia

**Keywords:** channel, FeoB, GTPase (guanosine 5′-triphosphatase), homology modelling, iron acquisition, membrane protein, pathogen

## Abstract

Iron is essential for the survival and virulence of pathogenic bacteria. The FeoB transporter allows the bacterial cell to acquire ferrous iron from its environment, making it an excellent drug target in intractable pathogens. The protein consists of an N-terminal GTP-binding domain and a C-terminal membrane domain. Despite the availability of X-ray crystal structures of the N-terminal domain, many aspects of the structure and function of FeoB remain unclear, such as the structure of the membrane domain, the oligomeric state of the protein, the molecular mechanism of iron transport, and how this is coupled to GTP hydrolysis at the N-terminal domain. In the present study, we describe the first homology model of FeoB. Due to the lack of sequence homology between FeoB and other transporters, the structures of four different proteins were used as templates to generate the homology model of full-length FeoB, which predicts a trimeric structure. We confirmed this trimeric structure by both blue-native-PAGE (BN-PAGE) and AFM. According to our model, the membrane domain of the trimeric protein forms a central pore lined by highly conserved cysteine residues. This pore aligns with a central pore in the N-terminal GTPase domain (G-domain) lined by aspartate residues. Biochemical analysis of FeoB from *Pseudomonas aeruginosa* further reveals a putative iron sensor domain that could connect GTP binding/hydrolysis to the opening of the pore. These results indicate that FeoB might not act as a transporter, but rather as a GTP-gated channel.

## Abstract (precis)

The bacterial ferrous iron acquisition protein FeoB assembles as a homotrimer that is predicted to form a central pore lined by conserved cysteine residues. Structure-function analysis of FeoB indicates a putative mechanism more akin to a GTP-gated channel than a transporter.

## INTRODUCTION

*Pseudomonas aeruginosa* is a ubiquitous Gram-negative opportunistic pathogen that is intrinsically resistant to multiple classes of antimicrobials [1,2]. It is associated with a range of life-threatening hospital-acquired infections, and is also the main cause of mortality in patients suffering from cystic fibrosis [3–5]. The isolation of clinical samples of *P. aeruginosa* resistant to a wide range of antimicrobials is increasing at an alarming rate. There is therefore an urgent need to discover new ways of treating these resistant infections [6–9].

The acquisition of iron is central to the survival of pathogens, as well as being essential for virulence and biofilm formation [10–14]. Hence, the targeting of bacterial iron acquisition could provide an effective way to counter drug-resistant organisms such as *P. aeruginosa*. Ferrous iron (Fe^2+^) is acquired via the FeoABC system [10,15,16]. In this system, FeoA is a small (8.3 kDa) cytosolic protein, containing an SH3-domain that could potentially activate FeoB. FeoB is a large (83 kDa) protein, with an N-terminal soluble domain and a C-terminal integral membrane domain [10]. FeoC is a small (8.7 kDa) cytosolic protein thought to function in the transcriptional regulation of FeoB expression. FeoB is the major component of the Feo system, and is likely to act as the Fe^2+^ permease [10,15,17]. FeoB has been implicated in the virulence of many pathogenic bacteria, such as *Helicobacter pylori, Legionella pneumophila, Campylobacter jejuni, Streptococcus suis, Francisella tularensis* and uropathogenic isolates of *Escherichia coli* [18–23], and it is known to be required for tissue colonization [24]. FeoB also has an important role in the survival of *P. aeruginosa* in the anaerobic environment of biofilms, which are typical of the chronic infections of the lungs of people suffering from cystic fibrosis, where iron is prevalent in the reduced Fe^2+^ state [25]. FeoB is also involved in Fe^2+^-mediated biofilm formation [26].

Although its key roles are widely recognized, our knowledge of FeoB is still far from complete. Studies have so far focused on the soluble N-terminal domain of FeoB (NFeoB), although many questions regarding the structure and the function of the membrane domain remain unanswered. In addition, it is unclear how GTP hydrolysis at the N-terminal domain is coupled to Fe^2+^ transport by the membrane domain.

**Figure 1:**
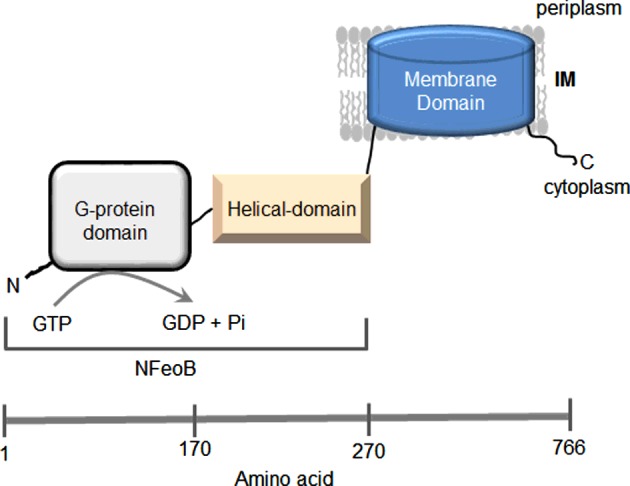
Schematic representation of the putative domain arrangement in FeoB FeoB consists of a G-domain (residues 1–170), a helical domain (residues 171–270) and a membrane domain (residues 270–766).

Structures of the N-terminal domain revealed two soluble domains: a GTPase domain (G-domain) and a five-helix domain (S-domain or helical domain) [27–31] (Figure 1). The structures of the G-domains are superimposable upon small eukaryotic G-proteins such as the human oncogene p21-Ras [30,32,33]. This domain has the five conserved sequence motifs (G1–G5) critical for nucleotide recognition and hydrolysis and two Switch regions (Switch I and Switch II) that undergo conformational changes in response to GTP binding and hydrolysis [27,34–38]. The oligomeric arrangement of FeoB is not clear from the reported NFeoB structures, as it was crystallized in three different oligomeric forms: monomers, dimers and trimers [27–31,35,36,39]. The isolated G-domain has a low affinity for GDP, but the presence of the helical domain increases the affinity of the G-domain for GDP without affecting its affinity for GTP [36]. For this reason, the helical domain has been proposed to act as a GDP-dissociation inhibitor (GDI) domain that stabilizes the GDP-bound state. It is also proposed that the helical domain responds to the hydrolysis of GTP by initiating structural changes in the membrane domain, thereby facilitating the uptake of Fe^2+^ [28,31]; however, structural analysis of NFeoB from *Gallionella capsiferriformans* revealed that a large portion of the helical domain was missing [40]. Hence, the exact role of the helical domain is still unclear.

The membrane domain is believed to act as a Fe^2+^ permease [10]. It is predicted to be an integral membrane domain with 8–10 membrane-spanning helices and two gate regions containing highly conserved cysteine residues [10,15,37].

In the absence of any structural information on the full-length protein, we constructed a homology model of FeoB. Subsequently, we conducted biophysical and biochemical measurements on FeoB from *P. aeruginosa*, which verified the predicted oligomeric state of FeoB. Functional analysis was performed to investigate the role of conserved cysteine residues in Fe^2+^ transport and from these results a putative mechanism that couples the G-domain and Fe^2+^ transport was constructed.

## MATERIALS AND METHODS

### Molecular modelling of FeoB

**Figure 2:**
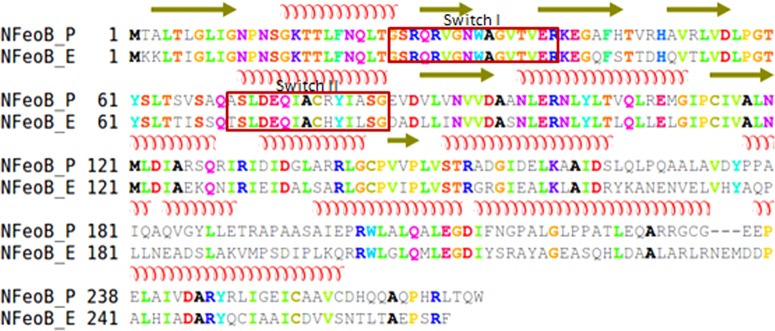
Alignment between NFeoB from *P. aeruginosa* (NFeoB_P) and NFeoB from *E. coli* (NFeoB_E) The secondary structures (α-helices and β-sheets) of NFeoB are shown above the sequences. The Switch regions are indicated in red boxes.

FeoB from *P. aeruginosa* (UniProt code: Q9HW43) was modelled in four stages. First, the modelling of the cytoplasmic domain of FeoB (NFeoB; residues 1–270), was based on the template structure of NFeoB from *E. coli* (PDB code: 3HYT) [27]. In this structure, NFeoB displays a trimeric stoichiometry when mantGTP, the non-hydrolysable GTP analogue, is bound. This template was selected because of a high level of identity (59%) between the FeoB proteins from *E. coli* and *P. aeruginosa* (Figure 2) and also because the structure was solved in the presence of MgSO_4_ and hence is more likely to be representative of the physiological state of FeoB. Using Modeller [41], 50 models of the liganded *E. coli* NFeoB protein containing the residues missing from the NFeoB structure, the mantGTP and Mg^2+^ were generated. The best model, with the lowest discrete optimized protein energy (DOPE) score, was chosen out of 200 options to represent the structure of the N-terminal G-domain of *P. aeruginosa*.

**Figure 3:**
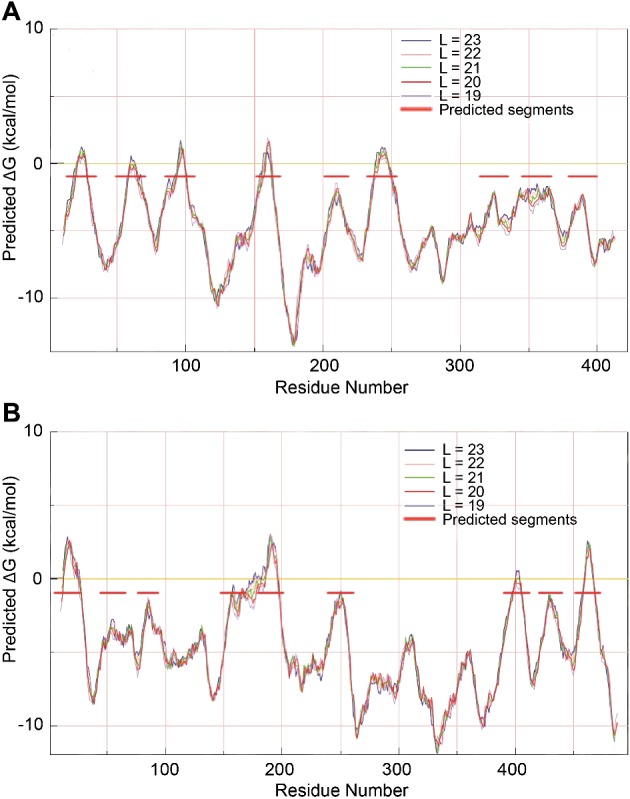
Prediction of the TM α-helices of the glutamate transporter, GltPh and FeoB Hydropathy plot of GltPh (**A**) and FeoB (**B**), according to translocon analysis by the Mpex programme. The positions of membrane-spanning α-helices are identified as peaks with horizontal red bars. *L* indicates the lengths of the sequence of residues in the model.

**Figure 4:**
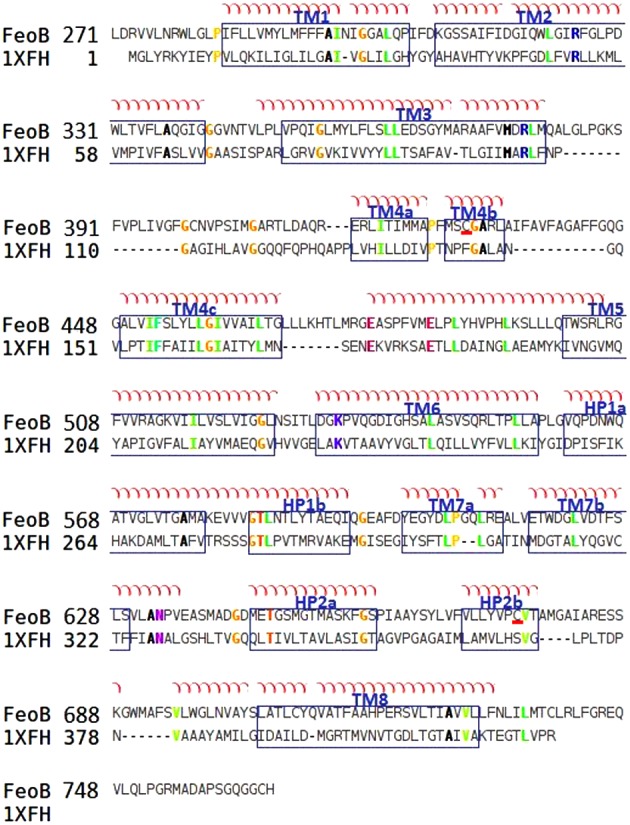
Alignment between the TM domains of FeoB from *P. aeruginosa* (FeoB) and of the glutamate transporter from *P. horikoshii* (1XFH) The TM segments of FeoB are arranged according to the crystal structure of the glutamate transporter and are visualized in blue boxes. The alignment was made using ClustalW and adjusted manually. Conserved cysteine residues are underlined in red.

Secondly, the transmembrane (TM) domain of FeoB (residues 271–766) was modelled based on its sequence similarity (22%) to the archaeal glutamate transporter from *Pyrococcus horikoshii*, GltPh (PDB code: 1XFH) [42]. Both proteins were predicted to contain nine TM helices by translocon-scale hydropathy plots [43] (Figure 3). Since the sequence similarity between FeoB and GltPh is relatively low for the use of standard methods, our approach used several methods to align the TM domains from the two proteins. A multiple sequence alignment of FeoB with other members of the GltPh transporter family was performed. Then, the alignment between the template and the TM domain of FeoB was manually corrected (Figure 4). One hundred models of the TM domain of FeoB were generated via Modeller using a slow refinement and optimization. The model with the best DOPE score was chosen.

Thirdly, a linker was chosen to connect the cytosolic NFeoB with the membrane domain of FeoB. To model this linker (residues 264–286), the EHD2 ATPase (PDB code: 2QPT) was used as template [44]. FeoB shares 19% identity with the sequence of the EHD2 ATPase, which is a dimer of 550 residues that plays a role in membrane reorganization in response to ATP hydrolysis [44]. Using the Pfam database, we found two Pfam matches for the sequence of EHD2 ATPase: one domain (residues 68–228) corresponds to dynamin_N of the dynamin family, and the other domain (residues 450–546) is the EF-hand_4 or cytoskeletal-regulatory complex EF hand. Dynamin is a large GTPase that is regulated by oligomerization and forms a collar-like structure around invaginations of membranes during the pinching-off process of vesicle formation [45]. The EH domain was subsequently found in several proteins implicated in endocytosis, vesicle transport and signal transduction in organisms ranging from yeast to mammals. Previously, more proteins have been demonstrated to have cation-dependent GTPase activity, such as dynamin proteins, where K^+^ ions stimulate GTPase activity [46]. The EHD ATPase protein is among the predicted cation-dependent GTPases from the dynamin superfamily [39]. The EHD proteins are distant members of this superfamily.

Finally, the C-terminal region of FeoB (residues 740–766) was modelled using the C-terminal domain of the dihydrodipicolinate reductase (PDB code: 1DIH) from the glyceraldehyde-3-phosphate dehydrogenase-like superfamily [47] as a template. FeoB shares 22% sequence identity with this enzyme.

### Protein production and purification

Growth of bacterial cells, preparation of inside-out vesicles and purification of histidine-tagged FeoB were performed as described recently [48].

### Blue-native PAGE

Blue-native-PAGE (BN-PAGE) was carried out using the NativePAGE™ Novex Bis–Tris Gel system (Invitrogen); a precast polyacrylamide system used for the separation of proteins in the non-denatured state. The system is based on the method described by Schagger and von Jagow [49], which uses Coomassie blue G-250 as the charge-shift molecule. This molecule binds to proteins and confers a net negative charge, while maintaining the proteins in the native state without any denaturation [50]. It is added to the samples containing non-ionic detergent prior to loading. It is also present in the cathode buffer to provide a continuous flow of Coomassie blue G-250 into the gel. BN-PAGE was performed at 140 V for 2 h using a 4%–12% Bis–Tris gel, according to the manufacturer's instructions. Upon completion of the run, the gels were placed in fix solution (40% v/v methanol and 10% v/v acetic acid) and microwaved at high voltage for 45 s, followed by incubation for 30 min at room temperature on a shaker. The fix solution was discarded and Coomassie Blue stain (0.1% w/v Coomassie Brilliant Blue R-250, 40% v/v methanol and 10% v/v glacial acetic acid) was added to the gel, followed by microwaving and overnight incubation at room temperature, with shaking. The gel was destained with destaining solution (8% v/v acetic acid) and microwaved again until clear enough for imaging.

### AFM

AFM imaging was carried out using a Bruker Multimode 2 instrument and a Nanoscope controller IIIa equipped with a 120 μm J-scanner and a dry imaging cell. Silicon cantilevers had a typical drive frequency of ∼300 kHz and a specified spring constant of ∼42 N/m (OTESPA, Bruker AFM Probes). The NV10 polymer used for the BN-PAGE was not compatible with AFM as it formed a sheet on the mica substrate. For this reason, C_12_E_8_ was used for the AFM measurements. We have recently shown that C_12_E_8_ is also a suitable detergent for FeoB [48]. Isolated FeoB was diluted 10000–20000 times in buffer [10 mM K-HEPES pH 7.0, 200 mM NaCl, 10 mM MgSO_4_, 0.05% C_12_E_8_ in ultra-pure (Biotechnology Performance Certified; BPC) water; Sigma–Aldrich] and 50 μl of the diluted protein was adsorbed on to freshly cleaved mica for 10 min. The sample was then washed with 10x 1 ml of BPC water and dried gently in a stream of nitrogen gas. After tuning and engaging of the AFM tip, 2×2 μm^2^ areas were scanned at 3–4 Hz. Further processing (flattening of images) and analysis of the observed particles (e.g. calculation of particle volumes) were done using the SPIP software (Image Metrology). The detection threshold was 0.25 nm and adjacent particles were split when gaps were below 3 nm. Particles with a molecular volume (Z Net Volume) below 50 nm^3^ were neglected. Molecular volume based on molecular mass was calculated using the equation: *V*_c_=(*M*_0_/*N*_0_)(*V*_1_ + *dV*_2_), where *M*_0_ is the molecular mass, *N*_0_ is Avogadro's number, *V*_1_ and *V*_2_ are the partial specific volumes of particle (0.74 cm^3^/g) and water (1 cm^3^/g) respectively, and *d* is the extent of protein hydration (taken as 0.4 g water/g protein).

It has been shown previously [51] that for a number of proteins the molecular volumes measured in air are very similar to the values obtained for proteins in fluid; hence, the process of drying does not significantly affect the measured molecular volume. It has also been shown by us [52] and Schneider et al. [51] that there is a close correspondence between the measured and predicted molecular volumes for various proteins over a wide range of molecular masses; hence, molecular volume is measured fairly accurately by AFM imaging in air.

### Generation of FeoB mutants

The C429S and C675S mutants of FeoB were generated by PCR with pFeoBH as a template, and using primers 5′-CGTTCATGTCCAGCGGCGCGCGCCTGGCGATC-3′ and 5′-GGCGCGCGCCGCTGGACATGAACGGCGCCATC-3′ for the C429S substitution and 5′-CTACGTGCCCAGCG-TGACCGCCATGGGCGC-3′ and 5′-GGCGGTCACGCT-GGGCACGTAGAGCAGGACG-3′ for the C675S substitution. PCR was performed using the high-fidelity Phusion DNA polymerase kit. The wild-type plasmid DNA template was removed by digestion of the PCR product with DpnI (New England Biolabs). The PCR products were digested with NdeI/HindIII (Fermentas) and ligated into digested pET41a(+) to yield plasmids pC429S-FeoBH and pC675S-FeoBH. The cloned PCR products were sequenced to ensure that only the intended changes were introduced.

### Measurement of GTPase activity

GTPase activity was determined with a malachite green assay [48,53] using the QuantiChrom™ GTPase assay kit (BioAssay Systems). The assay was based on the formation of a stable green-coloured complex between the malachite green and P_i_ released during hydrolysis of GTP. Reactions were prepared to give a final volume of 150 μl, containing the assay buffer (BioAssay Systems), 10 mM MgSO_4_, 1 mM GTP and 20–50 μg/ml of purified protein in 20 mM K-HEPES pH 7.0 buffer. The GTPase assay was performed at 37°C with continuous shaking after initiation of the reaction by addition of GTP (1 mM). Samples were taken at different time intervals over a total of 4 h. Reactions were terminated by addition of the colouring reagent (100 μl) and the *A*_630_ values were read after 30 min using a BioTek EL800 plate reader. A standard curve was prepared simultaneously using phosphate dilutions (0–5 nM).

## RESULTS

### Homology model of FeoB

So far, all structural and most functional studies on FeoB have focused on the soluble domain, NFeoB. However, in order to understand the molecular mechanism of iron transport and how this is linked to GTP hydrolysis, it is imperative to study the full-length protein. To this end, it would be extremely valuable to have a structure on which to base experimental design. However, progress in this field is hampered by the general difficulty in handling membrane proteins. Moreover, FeoB is such a unique type of transporter that it is difficult to find models that could act as a suitable blueprint.

**Figure 5:**
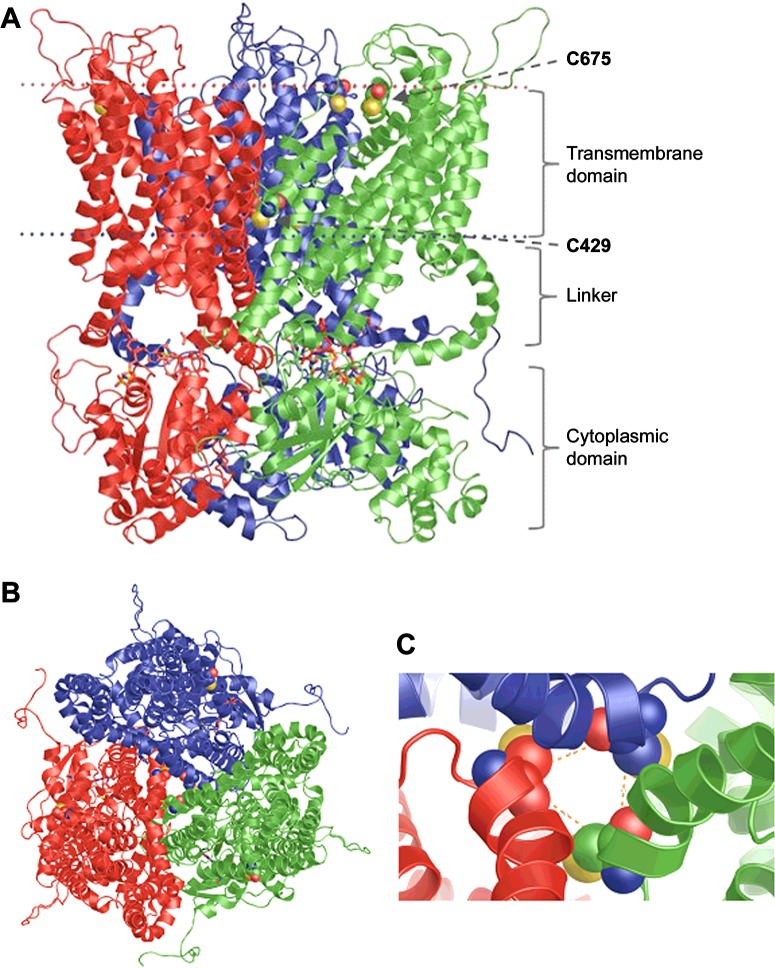
Homology model of FeoB from *P. aeruginosa* The three subunits are coloured green, red and blue. The conserved cysteine residues from TMH4 and TMH7 are drawn using space-filled atoms. The GTP ligands are in stick representation. The FeoB homotrimer is viewed either from the plane of the membrane (**A**) or and from the extracellular side of the membrane (**B**). TMH4s from the three monomers form a central pore. (**C**) Close-up view of the pore-forming TMH4s from the extracellular side of the membrane. The distances between the α-carbons of the TMH4 cysteine residues, indicated by orange dashed lines, are 8.5 Å.

Modelling of the cytoplasmic domain of FeoB (NFeoB; residues 1–270) was relatively straightforward owing to the existence of several crystal structures of this domain from various organisms. Due to the high level of identity (59%) between the FeoB proteins from *E. coli* and *P. aeruginosa*, our model of NFeoB was based on the template structure of *E. coli* NFeoB (PDB code: 3HYT), which was shown previously to crystallize as a homotrimer [27]. NFeoB exhibits the basic polypeptide fold of other prokaryotic and eukaryotic GTPases, consisting of a seven-stranded β-sheet surrounded by five α-helices (Figure 5). The consensus elements, G1–G4, which are involved in GTP and Mg^2+^ binding in all G-proteins, as well as the effector-binding regions recognized as Switch I (residues 25–40) and Switch II (residues 70–85), were identified based on the alignment shown in Figure 2. The *z*-DOPE score for our model was −0.26 whereas the RMSD value that compares the template and model was ∼3.6. The GA341 score (an indication for fold assessment) was 1.

In contrast with the cytosolic domain, it was difficult to find modelling templates for the TM domain of FeoB with traditional methods as there are no structures of membrane proteins with a high sequence similarity to FeoB. We therefore decided to use a protein with a related function, such as a membrane transport protein, which also contains motifs present in FeoB. As a result, we selected the glutamate transporter GltPh from *P. horikoshii* [42] which is also known to crystallize as a homotrimer (PDB code: 1XFH). Glutamate transporters exhibit an unusual topology, which includes the presence of two re-entrant loops. These transporters are the first examples of transport proteins that contain these features [54,55]. Unlike regular loops that connect membrane-spanning α-helices, re-entrant loops are not entirely extramembranous but instead enter into the membrane and help shape the membrane-embedded part of the protein.

Three algorithms were used to characterize the topology of the crystallized GltPh protein (TMpred, OCTOPUS, MPEx). Of these, only MPEx was able to predict TM helix (TMH) topology successfully, since MPEx was the only algorithm that identified the TMH4 and the TMH8 of the glutamate transporter (Figure 3). None of the algorithms used recognized the hairpin HP1. The translocon-scale hydropathy analysis by the MPEx software identifies TMHs based on translocon-mediated TMH assembly, considering amino acid position-dependent membrane insertion efficiency, as well as hydrophobic moment, TM segment length and flanking amino acid influences. The software calculates the free energy of translocon-guided TMH insertion into the membrane. So, MPEx was used to predict the topology of the TM domain of the FeoB transporter, which suggests that FeoB could have nine TMHs (including one hairpin) with N- and C-termini in the cytoplasm (Figure 4).

Upon further analysis of the GltPh transporter family (Pfam accession number: PF00375) in the Pfam database [56], certain motifs were revealed which incidentally were also present in FeoB. Among these were conserved cysteine residues located in TMH4 (Cys^429^) and TMH7 (Cys^675^). Cysteine residues are good ligands for soft Lewis acids, such as Fe^2+^, suggesting that the conserved cysteine residues in FeoB could be involved in metal binding during the transport process [10]. The predicted transport mechanism of our model highlights the location of Cys^429^ in the TMH4, suggesting that this helix may form the pore of the transporter. In the homotrimer, the three cysteine residues line a ring in the middle of the membrane domain with a diameter of 4.9 Å (1 Å=0.1 nm), which would represent an ‘open’ state and could allow Fe^2+^ to pass (Figure 5). The other cysteine is located at the top of TMH7 on the periplasmic face of the membrane.

Interestingly, the linker (residues 264–286) that connects the soluble domain to the membrane domain of FeoB could form an α-helix according to the sequence identity with the EHD ATPase protein (PDB code: 2QPT; residues 372–397). The space constraints derived from the modelling forced this helix into a semicircle; however, it is more likely to be a straight helix in the full-length protein once the GTP domain is active.

The C-terminal region of FeoB (residues 740–766) was modelled using as a template the C-terminal domain of the dihydrodipicolinate reductase from the glyceraldehyde-3-phosphate dehydrogenase-like superfamily [47] (PDB code 1DIH). The signature pattern found by the PROSITE server is the best conserved region in this enzyme. The motif DAPSG, which is located in the central section, is part of the substrate-binding region, and forms a buckle. Interestingly, this motif was also found in FeoB at residues 757–761. The C-terminal region of FeoB has been suggested to contain a potential Fe^2+^-binding cysteine/histidine-rich region, presumed to be located in the cytosol. This feature is somewhat similar to the metal-binding motifs (CXXC), which may have a chaperone function, found in the metal-transporting P-type ATPases [57]. This cysteine/histidine-rich region in FeoB may have a similar role, or could be more directly involved in the control of metal-dependent FeoB-activity control or Fe^2+^ translocation [10].

### Experimental testing of the predicted FeoB model

**Figure 6:**
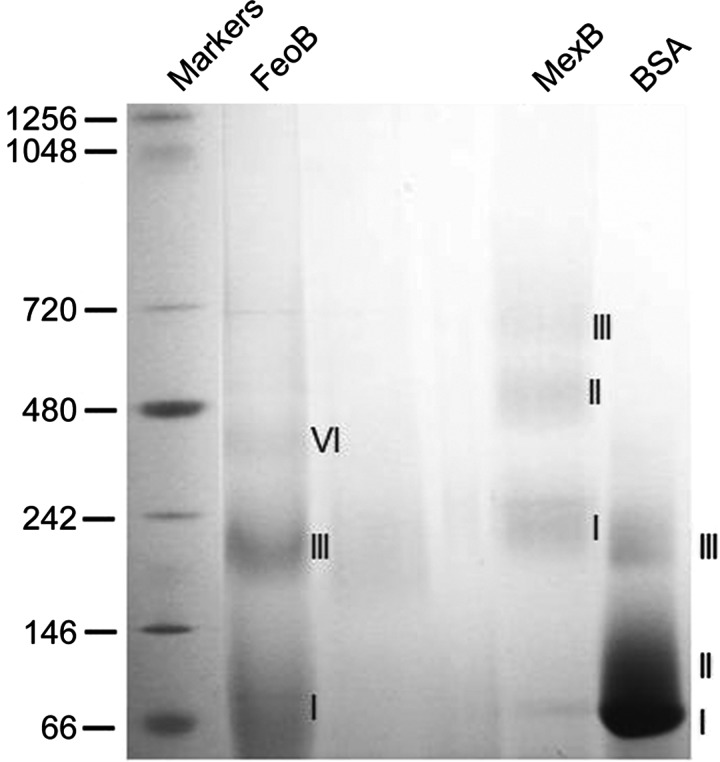
Oligomeric arrangement of FeoB. BN-PAGE of purified FeoB solubilized with the polymer NV10 For FeoB, bands I, III and VI probably correspond to monomer, trimer and hexamer. Bands I, II and III correspond to monomer, dimer and trimer for both MexB and BSA.

Recombinant FeoB was expressed in *E. coli* cells and purified as described recently [48]. The oligomeric state of purified FeoB in its native functional state in detergent was first assessed using BN-PAGE. The electrophoretic separation relies on binding of the dye Coomassie blue G-250 to proteins in exchange for the detergent, thereby overcoming the limitations of traditional native PAGE for membrane proteins [50]. In a recent study, we identified the polyfructose polymer NV10 as a suitable agent to preserve FeoB stability and function in solution [48]. When FeoB was purified in NV10, three distinct oligomeric states were observed, as shown in Figure 6. Based on the band migration pattern, these states most likely correspond to monomers, trimers and hexamers though it is not possible to be absolutely certain because detergent, lipid and variations in Coomassie blue G-250 binding may affect the migration pattern [58]. As a comparison, we included another integral membrane protein from *P. aeruginosa*, the drug efflux transporter MexB. MexB is a 112 kDa protein which is known to form trimers in detergent solution [59,60]. As expected, three distinct bands were also observed for MexB; however, in a manner typical of the behaviour of a membrane protein in BN-PAGE, the observed size of the oligomers did not correspond with the calculated sizes of dimers and trimers (Figure 6). BSA, a soluble protein, was also tested, and found to run as monomers, dimers and trimers of the expected sizes (Figure 6).

**Figure 7:**
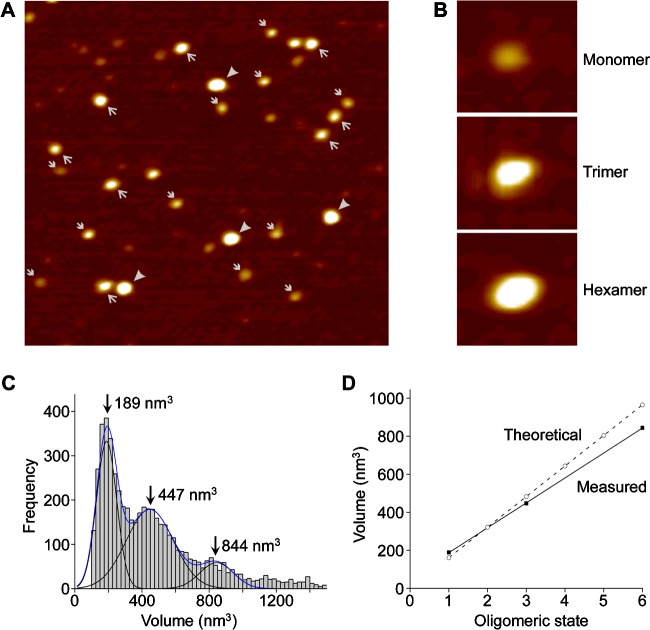
AFM demonstrates that FeoB assembles as a homotrimer in solution Histidine-tagged FeoB was solubilized and purified from inside out vesicles in buffer containing 0.05% C_12_E_8_. (**A**) Low-magnification AFM images of a sample of purified FeoB showing monomers (➔), trimers (→) and hexamers (➤). Images are 500 nm square. (**B**) Gallery of zoomed images showing examples of a FeoB monomer, trimer and hexamer. Images are 59 nm square. (**C**) Volume distribution of FeoB. The peak molecular volumes are indicated. (**D**) Relationship between FeoB volume and oligomeric state.

The assembly state of FeoB was further investigated at single-molecule resolution using AFM. AFM images of FeoB in 0.5% C_12_E_8_ indicated the presence of particles of various sizes (Figure 7A). More specifically, particles could be grouped into three size categories (illustrated in Figure 7B) that probably correspond to the three bands seen on BN-PAGE gels. A volume distribution of FeoB, generated from the AFM images, revealed populations of particles of molecular volumes 189 nm^3^, 447 nm^3^ and 844 nm^3^ (Figure 7C). As shown in Figure 7(D), there was a perfect linear fit of the measured volumes (*R*=0.99998). Figure 7(D) also shows the close correspondence between these volumes and the volumes expected for FeoB monomers, trimers and hexamers, supporting our conclusions based on the BN-PAGE data. Given the trimeric structure predicted by the modelling, we suggest that in detergent solution FeoB assembles as a trimer. The small volume peak probably represents monomers produced by disassembly of the trimers during isolation, whereas the large volume peak probably represents pairs of trimers. Note that in our recent and previous AFM studies of receptors and ion channels, we have seen similar examples of both disassembly [61] and aggregation of oligomeric proteins [62].

### Role of conserved cysteine residues

**Figure 8:**
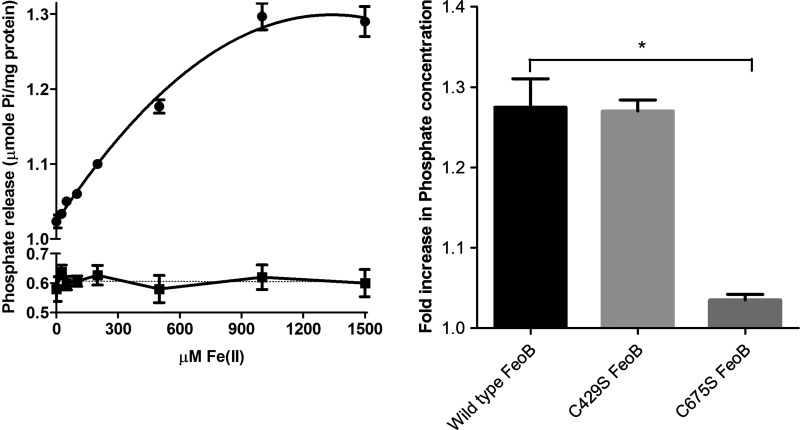
Effect of Fe^2+^ on the GTPase activity of FeoB (**A**) GTPase activity of wild-type FeoB (●) and D123N FeoB (■) was measured in the presence of varying concentrations of FeSO_4_ (0–1.5 mM). FeSO_4_ stimulated activity was observed with maximum stimulation at a FeSO_4_ concentration of 1 mM (*n*=6). (**B**) GTPase activity of wild-type FeoB and FeoB mutants (all 30 μg) was measured in the presence and absence of FeSO_4_ (1 mM). The fold increase in P_i_ concentration after 4 h between stimulated and unstimulated proteins was calculated; *P<0.005, compared with wild-type, *n*=4, one-way ANOVA.

In addition to predicting a homotrimeric structure, our model also identified two conserved cysteine residues which could be involved in Fe^2+^ transport. In order to investigate the roles of these residues, we first established a method for measuring the direct involvement of FeoB in Fe^2+^ transport. ATPases and GTPases normally have a low basal activity which is stimulated by their substrates. Given the predicted function of FeoB as a Fe^2+^ transporting protein, the GTPase activity of the protein was tested in the presence of Fe^2+^. To ensure that the reduced state of iron was maintained throughout the reaction, FeSO_4_ was prepared in ascorbic acid, and this reducing agent was also included in the reaction to prevent the oxidation of iron. The GTPase activity of FeoB was monitored in the presence of varying Fe^2+^ concentrations. As predicted, an increase in Fe^2+^ concentration caused a progressive increase in GTPase activity, up to a maximum at 1–1.5 mM Fe^2+^ (Figure 8A). In contrast, there was no increase in GTPase activity for the D123N mutant of FeoB upon the addition of Fe^2+^ (Figure 8A).

To determine if the conserved cysteine residues (Cys^429^ and Cys^675^) are involved in Fe^2+^ transport, mutants of FeoB were prepared in which the cysteine residues were replaced with serine residues. The GTPase activities of C426S FeoB and C675S FeoB were determined in the absence or presence of Fe^2+^. The basal GTPase activity of FeoB was not affected by either mutation (results not shown). As Cys^429^ lines a central pore in the derived structure, it was expected to play a central role in Fe^2+^ transport. Unexpectedly, the Fe^2+^-stimulated GTPase activity of C429S FeoB was not different from that of the wild-type protein (Figure 8B). In contrast, the stimulation of the activity of C675S FeoB by Fe^2+^ was significantly reduced, so that there was little stimulation above the basal level.

## DISCUSSION

Since 2009, several structures of the NFeoB from various organisms have been published. However, these advances have not yet been translated into mechanistic insights; moreover, the structure of full-length FeoB is still unavailable. In the present study, we present a model of full-length FeoB from *P. aeruginosa*. We also provide functional data which, combined with our model, suggests a putative mechanism of action of this intractable protein. It is generally believed that the TM domain of FeoB forms the pore for Fe^2+^ transport, whereas the G-domain regulates transport activity; however, this has not been established experimentally [63]. In our study, we demonstrate a Fe^2+^-stimulated GTPase activity of FeoB. This result represents the first evidence for a direct interaction of FeoB with ferrous iron; further, it provides a link between GTP hydrolysis at the NFeoB and substrate transport through the membrane domain.

Our model predicts a trimeric structure for FeoB. The assembly state of FeoB has been controversial, because of the variation between results obtained with different solutions, organisms and nucleotide-bound states. For instance, NFeoB from *E. coli* was found to assemble as a trimer in both apo- and nucleotide-bound forms [64]. However, a study of *Themotoga maritima* NFeoB revealed that apo-FeoB exists as a monomer in solution, whereas GDP- and GMP-PNP-bound FeoB behaves as a dimer [65]. In addition, a monomeric structure of FeoB was found in *L. pneumophila* [66], whereas a dimeric structure was observed in *Methanococcus jannaschii* [67], and NFeoB from *Klebsiella pneumoniae* crystallized as a trimer [68]. This same study also revealed binding of FeoC to FeoB, and showed that the binding of FeoC inhibited the trimerization of FeoB. Interestingly, the presence of Mg^2+^ seems to be a common factor in all trimeric assemblies of NFeoB, regardless of species.

The NFeoB is conserved, and the modelled structure was consistent with our expectations (Figures 3 and 5). In contrast, the model of the membrane domain was very surprising. In the absence of any structures of FeoB homologues, we used a protein of similar function, i.e. a glutamate transporter (GltPh), as a template for the membrane domain. Interestingly, it has been suggested that glutamate transporters combine transporter- and channel-like features [54]. The mechanism of these transporters is based on their ability to change between two states: outward-facing, where the substrate binding is accessible from the extracellular side, and inward-facing, where binding occurs from the cytoplasm. The 3D structure of GltPh was determined by X-ray crystallography [42], and revealed the presence of two re-entrant loops [55]. Re-entrant loops have also been observed in the crystal structures of the bacterial K^+^ channel KcsA and in the water- and glycerol-conducting channels of the aquaporin family, where the loops were shown to line aqueous TM pores through which the substrates flow [69–71]. The re-entrant loops in glutamate transporters are now known as re-entrant helical hairpins, which are considered to act as extracellular gates [42,72]. In spite of the low level of identity between FeoB and GltPh, our model indicated a trimeric structure, with eight TM helices per monomer (Figures 4 and 5). The trimer was arranged to form a central pore consisting of TMH4. Intriguingly, this pore was lined with three highly conserved Cys^429^ residues, one from each monomer. This cysteine residue is on the cytoplasmic face of the inner membrane and would be in Gate 1 (Figure 5). The pore shows significant similarities with the glutamic acid-lined cytoplasmic pore observed in the trimers of NFeoB from *E. coli* [27]. Our finding that the same pore also occurs in the membrane domain is remarkable and represents a very strong indication of the importance of Cys^429^ in transport. In our model, the N-terminal cytosolic domain of FeoB is also predicted to form a cytosolic pore lined by Asp^133^ residues (Glu^133^ in *E. coli*). The pore in NFeoB is aligned with the pore in the TM domain, so that it could potentially form a continuous pathway for Fe^2+^. In NFeoB from *E. coli*, the nucleotide-free and nucleotide-bound structures correspond to the closed and open states of a central cytoplasmic pore respectively [27]. The 4.5 Å diameter of the pore in our model would correspond to the open state of the pore. Note also that a similar cytoplasmic aspartic acid-lined pore has also been found in CorA, a pentameric transporter for divalent metal ions such as Mg^2+^ [73].

**Figure 9:**
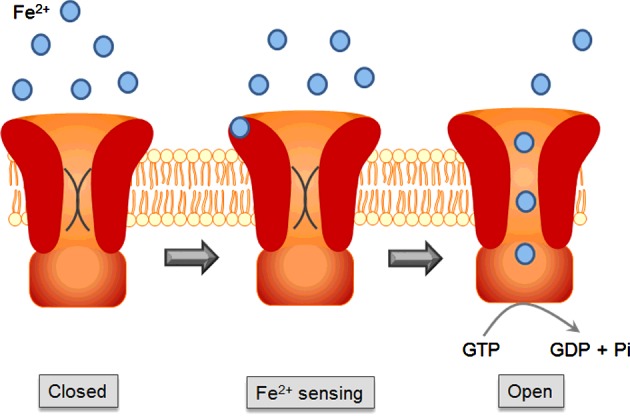
Schematic representation of the putative mechanism of action of FeoB from *P. aeruginosa* acting as a GTP-gated Fe^2+^ channel Fe^2+^ in the periplasm binds to Cys^675^, which acts as a Fe^2+^ sensor. Fe^2+^ binding signals the G-domain to initiate the binding of GTP. Either GTP binding or GTP hydrolysis results in conformational changes that subsequently open the pore lined by Cys^429^, allowing Fe^2+^ to pass down its concentration gradient.

The second conserved cysteine residue (Cys^675^) was positioned on TMH7 at the membrane-periplasm interface in Gate 2 (Figure 5). Unlike Cys^429^, Cys^675^ did not line a pore in our model, but was positioned on the lipid-facing side of the seventh TM helix; hence. it was not obvious how this residue could play a part in Fe^2+^ transport. The results of the Fe^2+^-stimulated GTPase assays were therefore quite unexpected. Mutating Cys^675^ severely affected substrate-stimulated GTPase activity, whereas mutation of C429S had no effect. A possible explanation of these results could be that Cys^675^ acts as a Fe^2+^ sensor, signalling to the G-domain to initiate the binding of GTP, which would then cause the pore lined by Cys^429^ to open and allow the passage of Fe^2+^. According to this idea, a mutation in the ‘recognition’ motif (Cys^675^) would hamper Fe^2+^-stimulated GTPase activity, whereas a mutation in the ‘pore’ motif (Cys^429^) would only affect substrate transport. Figure 9 provides a schematic diagram for a putative mechanism of action that would connect the model with the observed Fe^2+^-stimulated GTPase activity.

FeoB has always presented a conundrum. The G-domain is needed for Fe^2+^ transport; however, the GTP hydrolysis rate is too low to drive active transport. Many small GTPases such as Ras display the same low rates of GTP hydrolysis, which can be accelerated by GTPase-activating proteins (GAPs). It has been suggested that FeoA, the small cytosolic protein from the Feo operon, could act as a GAP to activate FeoB [10]. FeoA shares a minor degree of sequence homology with DtxR (diphtheria toxin regulator protein), which functions as an iron-responsive protein in *Corynebacterium diphtheria*. Further, FeoA contains an SH3 domain, which would typically mediate protein–protein interactions [10,74]. This suggests a potential stimulatory role of FeoA on NFeoB activity [74–76]. However, we were unable to observe any stimulation of GTPase activity by FeoA (results not shown), consistent with the same finding based on the use of NMR [75]. The GTPase activity of NFeoB from *E. coli* could be stimulated in part by K^+^ ions [75,77]; however, such stimulation has not been observed for FeoB from *P. aeruginosa* (results not shown).

At present the most plausible mechanism of FeoB action, which is compatible with the biochemical measurements, is that FeoB does not act as a transporter, but rather as a GTP-gated channel, just as ATP hydrolysis drives gating in the cystic fibrosis transmembrane conductance regulator (CFTR). Similar to FeoB, CFTR also has a very low nucleotide hydrolysis rate that is only loosely connected with channel activity [78]. In fact, the prokaryotic homologue of CFTR does not act as a channel but rather as a Cl^−^/H^+^ exchange transporter, indicating that the boundary between channels and transporters is not clear cut [79]. Whether FeoB acts as GTP-driven transporter, a GTP-gated channel or a Fe^2+^/H^+^ antiporter still needs to be established. The placement of the residues in our model also still has to be verified experimentally. However, the notion of sensor and pore domains in a GTPase is completely novel. Our model not only provides the first structure of full-length FeoB, but also predicts many interesting new features of the protein. We suggest that use of this model could inform many future experiments into the structure and molecular mechanism of FeoB proteins.

## Abbreviations

BN-PAGE: blue-native-PAGE
BPC: Biotechnology Performance Certified
CFTR: cystic fibrosis transmembrane conductance regulator
DOPE: discrete optimized protein energy
GAP: GTPase-activating protein
G-domain: GTPase domain
NFeoB: N-terminal domain of FeoB
PDB: protein data bank
TM: transmembrane
TMH: transmembrane helix
